# The Experimental Comparison of Abrasion Resistance of Extruded and 3D Printed Plastics

**DOI:** 10.3390/ma18071592

**Published:** 2025-04-01

**Authors:** Maciej Kujawa, Anita Ptak

**Affiliations:** Department of Fundamentals of Machine Design and Mechatronic Systems, Faculty of Mechanical Engineering, Wroclaw University of Science and Technology, 50-371 Wroclaw, Poland; anita.ptak@pwr.edu.pl

**Keywords:** material testing, tribological properties, abrasive wear, hardness, surface topology, FDM, FFF

## Abstract

3D printing is becoming widely used and printed parts very often replace extruded parts. Plastics, due to their ability to work with steel without lubrication, are commonly used for sliding components and are therefore exposed to various types of wear, including abrasive wear. In this paper, abrasive wear resistance tests were carried out to compare extruded and 3D-printed samples. Moreover, microhardness tests, surface topography and microscopic observations of the surface of the samples before and after friction were also conducted. Samples were made from eight materials that are most commonly used in 3D FDM printing: PLA, PET-G, ABS, PA, PP, PC, PMMA and HIPS. For six out of the eight materials tested, samples made by extrusion proved to be more resistant to abrasive wear (between 10% and 24%) than those printed ones. Fabrication by 3D printing can lead to different object properties and thus different abrasion resistance. The abrasion resistance of extruded samples depends on factors reported in the literature such as hardness, density and surface roughness. In the case of 3D printed samples, no such relationship was found. For this reason, the researchers believe that the reduced abrasion wear resistance of printed samples is due to their specific internal structure.

## 1. Introduction

The global 3D printing market is expected to register a compound annual growth rate of 23.5% from 2024 to 2030 [1]. This indicates that 3D printing is constantly being used more widely. 3D printing is usually not associated with manufacturing, since in many cases it is more time-consuming than conventional technologies. In addition, there are many problems and difficulties in 3D printing [2]. Prints often fail due to nozzle clogging or the print peeling off. Sometimes, the quality and accuracy of prints are insufficient. Despite this, in some areas of manufacturing 3D printing offers significant benefits. Verhoef et al. [3] indicate that the use of 3D printing allows for shortening supply chains and, due to this, energy savings can amount to up to 25%. In addition, for conventional manufacturing technologies such as milling, up to 90% of material is lost during machining, whereas in 3D printing there is no such material loss [4]. Moreover, the use of 3D printing for production allows for obtaining components of between 5% and 95% lighter weight than their traditionally manufactured parts. It is worth noting that traditional manufacturing methods (injection moulding or die casting) require tooling, and tooling costs can be over 80% of total production costs [5,6].

3D FDM (Fused Deposition Modelling) printing is one of the cheapest and easiest methods of 3D printing. Even though this method does not require expensive and advanced equipment, it allows us to produce various elements with customised geometries at a low cost [7]. For instance, thanks to 3D FDM printing the production bench equipment which is tailored to the component being processed can be created [8]. 3D FDM printing is also commonly used to produce fixtures/mounts for various types of processes [9,10,11]. In this type of usage, printed components are very often exposed to abrasion. Furthermore, the printed components may come into direct contact with abrasive material [12]. Thus, it would be valuable to know what material used in 3D printing provides good abrasion resistance.

Due to the great popularity of FDM 3D printing, research work related to it is still being carried out. Above all, new materials are being implemented, as well as the composites based on thermoplastic polymers already used in 3D FDM printing. These are characterised by an increased thermal conductivity [13] or biodegradability [14,15,16,17]. What is more, much attention is being given to the 3D printing process itself and its improvement [18,19]. An important issue is also the determination of properties of 3D prints to apply printed components successfully in new areas. This can include, for example, testing the tribological properties of 3D FDM prints so that they can work with other materials.

The tribological properties of 3D FDM prints are mainly studied to determine the coefficient of friction and wear during sliding on steel [20,21,22,23,24,25,26,27]. It is possible to find papers presenting the abrasion resistance of 3D prints; however, there are limited studies about this issue. Abrasion resistance tests are often aimed at deciding whether 3D prints are suitable for specific applications, e.g., shoe heels or shoe bottoms [28], or foundry models [29]. There are also studies that aim to analyse the abrasion process of 3D prints. Suresha et al. [30] introduced Short Carbon Fibers (SCFs) to polylactide (PLA) and acrylonitrile butadiene styrene (ABS). However, the effects of SCFs on the abrasive wear of PLA and ABS composites proved to be not always positive. The mentioned studies concern only the two most popular materials in FDM 3D printing, i.e., PLA and ABS. Furthermore, no comparison of abrasion resistance of printed and extruded materials was found in the literature.

In the literature, analyses of the abrasion process of various materials with a solid structure (e.g., extruded or cast) can be found; however, there is little information about this process for various materials with a structure specific to FDM 3D printing. For this reason, the abrasion resistance of components made from the same material but with different technologies—3D printing and extrusion—was compared. The abrasion resistance was determined for the eight most commonly used materials in 3D FDM printing. In this way, it can be determined whether the statements formulated for a solid structure are suitable for a 3D FDM printing structure.

## 2. Materials and Methods

Wear resistance was measured during the tests. Wear resistance is influenced by, among other things: the hardness of the material, its stiffness, or the condition of the surface [31]. In view of this, the research was extended to include measurements of hardness and Young’s modulus, as well as an assessment of the surface of the samples using a microscope and profilometer. The specimens studied were made using FDM 3D printing technology and cut from extruded plates.

### 2.1. Preparation of Samples

**Figure 1:**
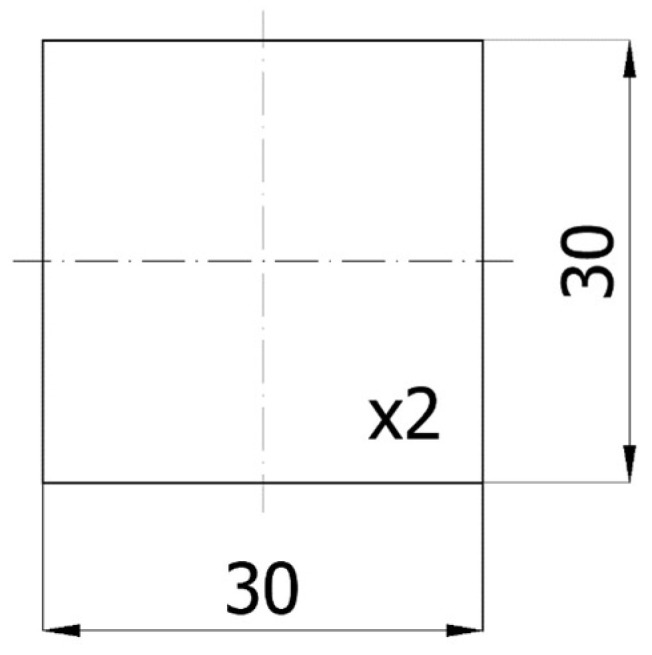
Dimensions of the abrasion resistance test samples.

Samples were made according to the guidelines of GOST 23.208-79 standard. The samples were cuboids with dimensions of 30 mm × 30 mm × 2 mm (width × length × thickness) (Figure 1).

#### 2.1.1. 3D Printed Samples

The samples were made on a 3D printer working in FDM technology: the Zortrax M300 Dual (Olsztyn, Poland). The machine is equipped with a heated bed and a closed chamber (without heating the air in the chamber). Samples were printed from eight materials that are most commonly used in FDM 3D printing [22] (Table 1): polylactide (PLA), polyethylene terephthalate glycol-modified (PET-G), acrylonitrile butadiene styrene (ABS), polyamide 12 (PA12), polypropylene (PP), polycarbonate (PC), poly (methyl methacrylate) (PMMA) and high-impact polystyrene (HIPS). Filaments of the first four materials (PLA, PET-G, ABS and PA12) were purchased from the printer manufacturer (Zortrax, Olsztyn, Poland); the next (PC, PMMA, HIPS) were supplied by F3D Filament (Finnotech, Katowice, Poland), and the polypropylene (PP) filament, which was not available from the aforementioned manufacturers, came from the manufacturer Fiberlogy, Brzezie, Poland. The filaments which were used had a diameter of 1.75 mm. The most important material processing parameters for printing, i.e., nozzle temperature and bed temperature, are shown in Table 1.

**Figure 2:**
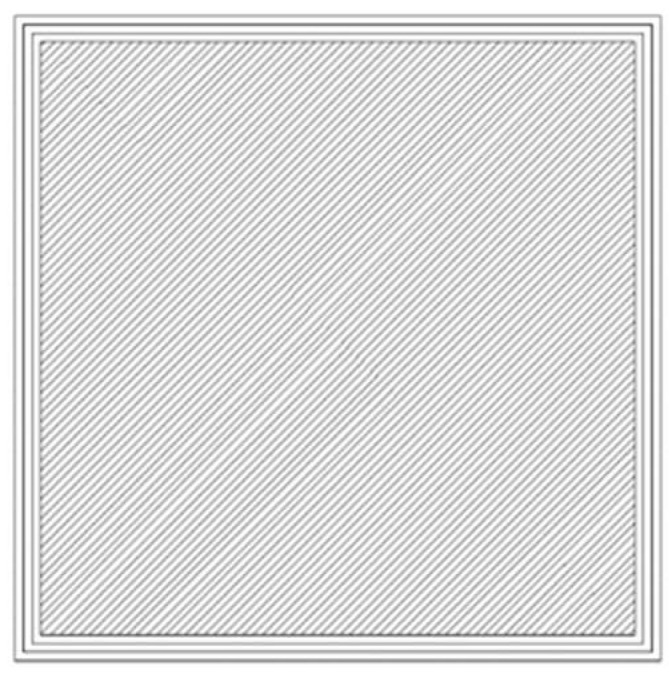
The method of laying material paths (filling at 45° to the edge of the sample and triple contour).

The 3D model of the sample was uploaded to Z-SUITE software version 3.5.0 (software provided by the printer manufacturer Zortrax, Olsztyn, Poland). With the help of this software, printer control files were obtained. The key parameters for the application of the material path of printing process are listed in Table 2. The nozzle diameter, layer height and number of contour paths corresponded to the most commonly used parameters during 3D FDM printing. Infill was also laid out in the most common way at an angle of 45° to the edge of the sample (Figure 2) (the fill of the next layer was laid out at an angle of 90° to the fill of the previous layer). The density of the infill was set at 100% (full infill) and no space was derived between the contour and the infill, so as to achieve a large infill and a strong merging of the infill with the contour. It appears that this will be beneficial in terms of abrasion resistance.

#### 2.1.2. Samples Cut from Extruded Plates

Due to the time-consuming nature of the 3D printing process, it is mainly used for one-off or short-run production. The study was designed to provide information on whether it is more advantageous to produce a component using a 3D printer or to use conventional manufacturing methods, taking into account abrasion resistance. Conventional manufacturing methods are understood as the use of prefabricated plastic parts and their further processing and assembly. Prefabricated plastic parts are mainly obtained by extrusion. For this reason, the test specimens were cut from extruded sheets. Plates with a thickness of 2 mm were obtained from the Polish representative office of Plastics International. The samples cut from the plates had the same dimensions as the printed samples (30 mm × 30 mm × 2 mm—Figure 1). The same set of materials was used as for the printed samples shown in Table 1 (except for polyamide—explained in the next paragraph).

In the case of polyamide, polyamide 12 was used for the printed samples and polyamide 6 for the samples cut from extruded plates. These materials belong to the same group; however, they differ in chemical structure and properties.

When producing sheets, PA6 is overwhelmingly used, whereas sheets made of PA12 are rare. When producing filaments for FDM 3D printers, the opposite is true: PA12 is by far the predominant material and PA6 is rare.

It was decided to compare samples printed from PA12 against samples extruded from PA6, as the main aim of the research was to indicate whether 3D printing or prefabricated parts are better to use for the production of a component. Such a comparison will be more beneficial for 3D printer users, as it will help them decide whether to purchase a filament or a prefabricated part. Comparing the same grades of polyamide (PA6 sheet versus PA6 print and PA12 sheet versus PA12 print) would have made the research more scientific, but would have deprived it of the practical conclusions that the authors cared more about.

### 2.2. Abrasion Resistance Measurement

**Figure 3:**
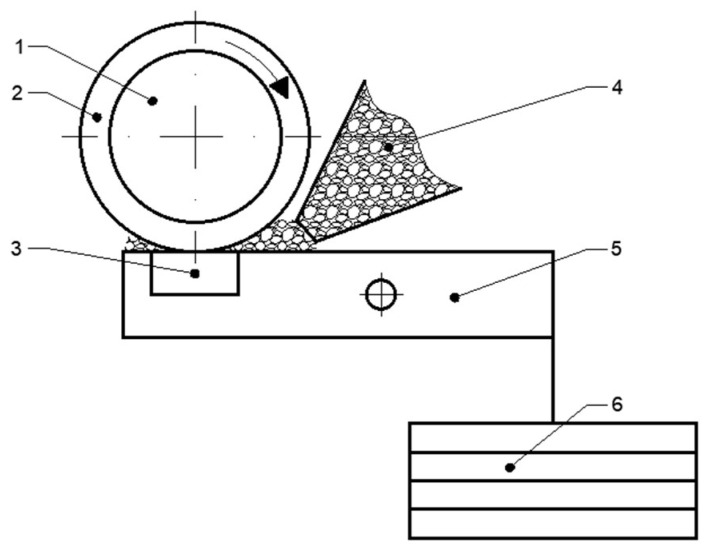
Scheme of the abrasive wear resistance test apparatus: 1—steel disc, 2—rubber rim, 3—sample, 4—nozzle with electrocorundum, 5—lever, 6—weights.

Wear resistance was measured using a T-07 tester manufactured by MCNEMiT in Radom, Poland. This device carries out wear resistance tests according to the guidelines contained in GOST 23.208-79 standard. The described method of testing is very similar to the more widely used ASTM G65-16 standard. An abrasive is continuously introduced between a rotating steel disc with a rubber rim and the sample (Figure 3). An abrasive was an electrocorundum indicated as 90. An electrocorundum is a synthetically produced form of aluminium oxide Al_2_O_3_ with regular and sharp crystals. The indication 90 states that the grain size of the abrasive ranged from 125 to 180 µm (according to PN-76/M-59115 standard).

The abrasive interacted with the sample causing it to lose mass. The mass loss was determined by weighing the sample before and after the test. However, the results were presented as a volume loss. The volume of material removed was calculated using the change in material mass and density. The material density of the sample was obtained by measuring its mass and dimensions (the volume of the sample was calculated from the dimensions).

Detailed apparatus and measurement parameters are included in Table 3.

### 2.3. Microhardness

An HMV-2 microhardness tester (Shimadzu, Kyoto, Japan) was used during the research. For the Vickers microhardness measurements, the lowest load (98.07 mN) and the lowest load time (5 s) were used. The 3D printed PP was so flexible that no imprint was observed on it using the Vickers method. For this reason, the hardness of the PP was measured with a Sauter HDD 100-1 hardness tester showing values on the Shore D hardness scale. The Shore method involves determining the depth to which the intender penetrated the material. The Vickers method involves making an imprint with the pyramid-shaped intender, measuring the imprint’s diagonal and calculating the hardness (*HV*) from the Formula:

(1) $$HV=\frac{F}{A}=\frac{F*2\sin\left(136{}^{\circ}/2\right)}{d^{2}}$$

where:

*F*—sample size the force applied to the intender;

*A*—the surface area of the imprint;

*d*—diagonal length of the imprint;

*136°—*angle between opposite faces of a pyramid-shaped intender.

### 2.4. Young’s Modulus

**Figure 4:**
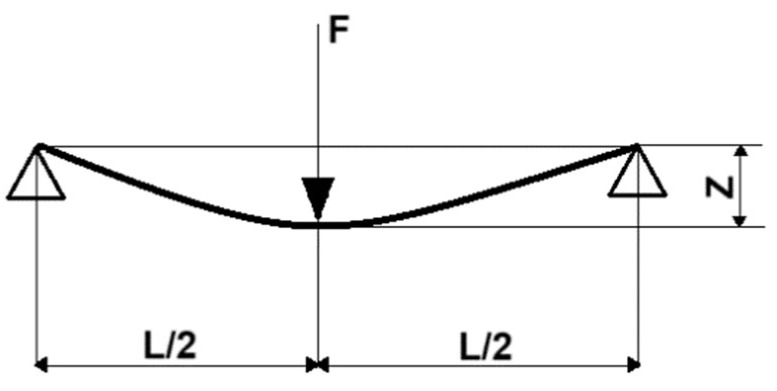
Scheme of calculation of the Young’s modulus using the three-point method for bending.

The calculation of the Young’s modulus (*E*) was made using the three-point method for bending on the basis of the PN-82/C-89051 standard (Figure 4). The samples made for testing had the shape of 10 mm × 4 mm beams. During the tests, the sample was evenly loaded to a maximum force equal to 5 N, the load was maintained for 20 s, and then the sample was evenly unloaded until the load was completely removed. The value of the Young’s modulus was calculated using mathematical Formula (2).

(2) $$E=\frac{FL^{3}}{zbh^{3}}$$

where:

*F*—force applied to the beam;

*L*—distance between supports;

*z*—deflection;

*b*—beam width;

*h*—beam height.

### 2.5. Sample Surface Evaluation

The surface parameters of the samples were evaluated using various methods. A ZEISS Discovery V12 optical microscope (Carl Zeiss, Jena, Germany) and SEM Phenom PRO X (ThermoFisher Scientific, Waltham, MA, USA) were used to assess the surface of the studied materials after tribological tests. A backscattered electron detector working in the topographic mode was used during the examinations of the SEM microscope at the accelerating voltage of 15 kV. A Leica DCM8 3D profilometer (Leica Microsystems, Wetzlar, Germany) was used to analyse the topography and quantify of the wear after testing. To describe the roughness of the surface, a Sa parameter (arithmetic mean height) was used since it provides a comprehensive representation of the overall surface topography. Sa is not sensitive to the occurrence of isolated peaks or valleys, as is the case with other parameters such as Sz or Sp. This is particularly important in abrasive processes, where material removal occurs uniformly over the surface. Furthermore, Sa is widely used in characterizing surfaces machined by abrasive techniques, which facilitates comparison with results presented in the literature.

### 2.6. Statistical Analysis

When presenting the results, the average value and the expanded uncertainty were given. The expanded uncertainty (*U*) was calculated using Formula (3):

(3) $$U=\frac{kS}{\sqrt{n}}$$

where:

*k*—coverage factor (*k* = 2);

*S*—standard deviation;

*n*—number of measurements.

In order to determine the relationship between two properties (e.g., volume loss and hardness), the Pearson correlation coefficient (*PCC*) was calculated using the following equation:

(4) $$PCC\left(X,Y\right)=\frac{\sum_{i=1}^{n}\left(x_{i}-\bar{x}\right)\left(y_{i}-\bar{y}\right)}{\sqrt{\sum_{i=1}^{n}{\left(x_{i}-\bar{x}\right)}^{2}}\sqrt{\sum_{i=1}^{n}{\left(y_{i}-\bar{y}\right)}^{2}}}$$

where:

*n*—the sample size;

*x*—the individual sample points indexed with *i*;

$\bar{x}—$the sample mean;

*y*—the individual sample points indexed with *i*;

$\bar{y}\;$—the sample mean.

When the obtained results were compared, the percentage difference between them was given. The percentage differences (Δ) were calculated taking into account the average values of the measured values. Additionally, the minimum (Δ_min_) and maximum (Δ_max_) percentage differences were calculated. The obtained expanded uncertainty ranges were used for this purpose. The maximum percentage difference was given as the ratio of the average value increased by the expanded uncertainty value to the average value reduced by the expanded uncertainty value. The minimum percentage difference was given as the ratio of the average value reduced by the expanded uncertainty value to the average value increased by the expanded uncertainty value. The following formulas were used for these calculations Equations (5)–(7):

(5) $$\Delta =\frac{\bar{x}-\bar{y}}{\bar{y}}$$

where:

$\bar{x}$—the sample mean;

$\bar{y}$—the sample mean.

(6) $$\Delta_{min}=\frac{\left(\bar{x}-U\right)-\left(\bar{y}+U\right)\;}{\bar{y}+U}$$

where:

$\bar{x}—$the sample mean;

$\bar{y}\;$—the sample mean;

*U—*the expanded uncertainty.

(7) $$\Delta_{max}=\frac{\left(\bar{x}+U\right)-\left(\bar{y}-U\right)\;}{\bar{y}-U}$$

where:

$\bar{x}$—the sample mean;

$\bar{y}\;$—the sample mean;

*U*—the expanded uncertainty.

The percentage difference between the average values is given before the brackets. The range in which the percentage difference is located with the measurement uncertainty taken into account is given within the brackets. Analysis of the given ranges allowed us to find out if the observed differences between the examined samples are statistically significant. This illustrates whether for each value in the expanded uncertainty range the compared results are larger, smaller, or there is no certainty about this since observed differences among the examined samples are statistically insignificant.

## 3. Results

The chapter presents the results of abrasion resistance, hardness and Young’s modulus tests. In addition, the surface of the specimens is presented using an SEM microscope and profilometer.

### 3.1. Abrasion Resistance

**Figure 5:**
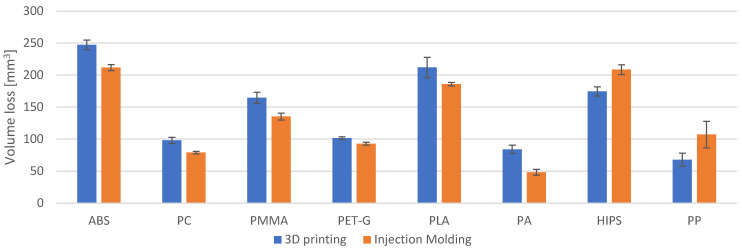
Volume loss obtained during the abrasion resistance testing.

For six out of the eight samples tested, the extruded materials proved to be more resistant to abrasive wear than the printed ones (Figure 5). In the majority of cases (Table 4), the extruded samples had an abrasion wear resistance higher by about 10% (PET-G) to 20% (PC). The results obtained for PP, PA and HIPS break out of this trend. For PP and HIPS, the extruded sample had a lower abrasion wear resistance (by about 34% for PP and 16% for HIPS). For PA, the extruded sample was 75% more resistant to abrasive wear.

**Figure 6:**
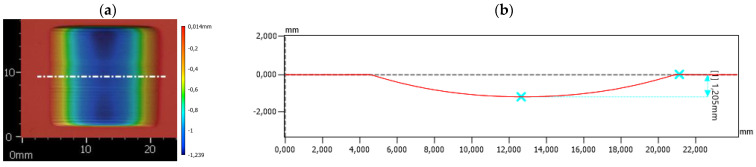
The measurement of the depth of the wear scar: (**a**) the position of the plane in which the profile was determined (marked by a white dash dot line—in the middle of the width of the wear scar); (**b**) sample profile and obtained depth.

In addition, the wear scar depth at half its width was determined using a profilometer (Figure 6). The depth of the wear scar is not constant over its entire width. What is more, the profile obtained halfway across the width of the wear scar will be slightly different to the profile obtained near the edge of the wear scar. For this reason, the volume loss (Figure 5) is the most reliable. Thus, the analysis presented in this paper was based on the volume wear results. Nevertheless, the depth of the wear scar also provided valuable information.

**Figure 7:**
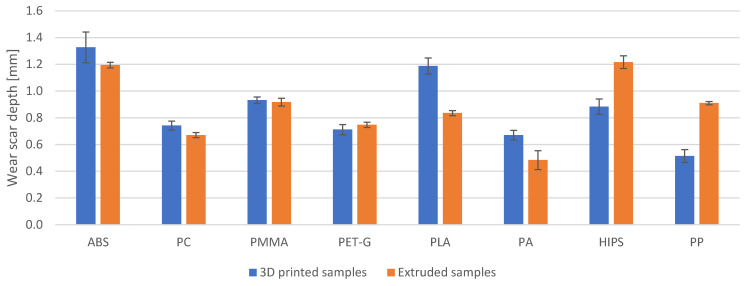
Wear scar depth measured after the abrasion resistance testing.

The obtained wear scar depths are shown in Figure 7, whereas the percentage differences in wear scar depth for the printed and extruded samples are given in Table 5. While comparing the changes in volume loss and wear scar depth with each other (Table 4 and Table 5), it can be seen that they mostly differ by a few percentage points. This is most likely due to the fact that the wear scar is not a perfect section of the cylinder (as described in the paragraph above).

**Figure 8:**
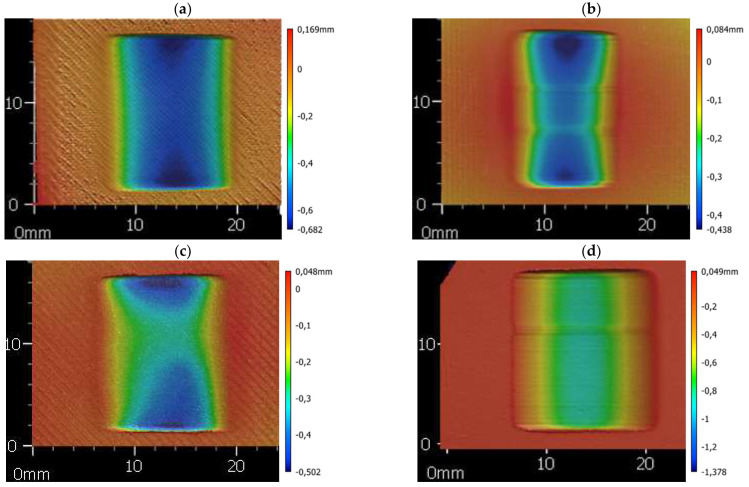
The 3D measurement of the wear scar: (**a**) 3D printed PA; (**b**) extruded PA; (**c**) 3D printed PP; (**d**) extruded PP.

Furthermore, the values obtained for the PA and PP appear to be worth mentioning. Namely, the differences for these materials are 46.8 percentage points for PA and 43.1 for PP. Such significant differences indicate that the shape of the wear scar deviates significantly from a portion of a cylindrical surface with a circular cross-section. The 3D scans from the profilometer confirm this phenomenon (Figure 8). Such a different geometry of the wear scar is most likely due to the different properties of PA and PP when printed and extruded. The other materials do not show such significantly different wear scar geometries.

### 3.2. Density

**Figure 9:**
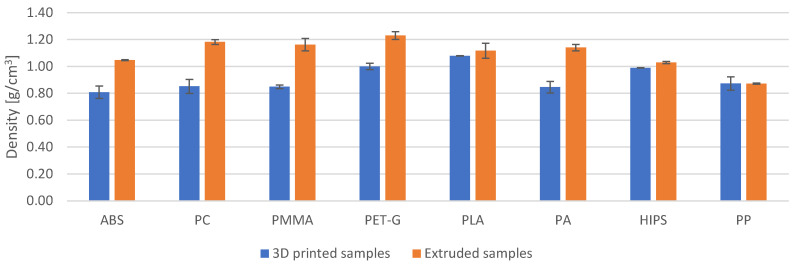
Density of samples made by extrusion and 3D printing.

Density calculations of the printed and extruded samples were also performed during the study. The density was calculated from the measured weight and dimensions of the sample. The results are shown in Figure 9 and Table 6. The density of the printed samples was lower for seven of the eight materials tested. Only for the PP was there no difference in density between the printed and extruded sample. For the ABS, PC, PMMA, PA and PET-G, the differences are significant and are in the range of 23% (PET-G) to almost 39% (PC). For the PLA and HIPS, the differences are minor at 3.5% and 4%, respectively.

The correlation coefficient between volume loss and density for extruded samples was 0.73, while for printed samples it was only 0.17. It should be concluded that for extruded samples there is a clear correlation, while for printed samples there is no correlation between density and abrasion resistance.

### 3.3. Hardness

**Figure 10:**
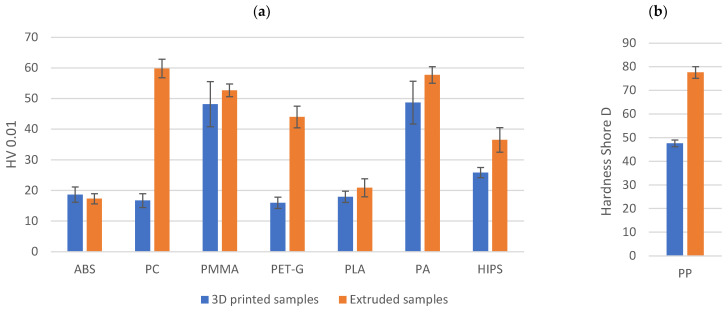
Hardness of the extruded and the 3D printed samples: (**a**) Vickers microhardness; (**b**) Shore D.

Microhardness measurements show that the majority of the extruded samples had a higher microhardness than the printed ones (Figure 10). The differences in values ranged from about 9% to as much as 258% (Table 7). The exception was ABS, for which the extruded samples had a microhardness about 7% lower than printed samples. The correlation coefficient between volume loss and microhardness for the extruded samples was 0.62, while for the printed samples it was only 0.08. It should be concluded that for the extruded samples there is a clear correlation between microhardness and abrasion resistance, whereas for the printed samples there is no such correlation. In the case of printed polypropylene, the material was so flexible that it was not possible to obtain an imprint on the surface and measure the microhardness. For this reason, the hardness of the PP is given on the Shore D hardness scale (Figure 10b). The extruded PP sample is 1.63 times harder than the 3D printed sample.

### 3.4. Surface Topography

**Figure 11:**
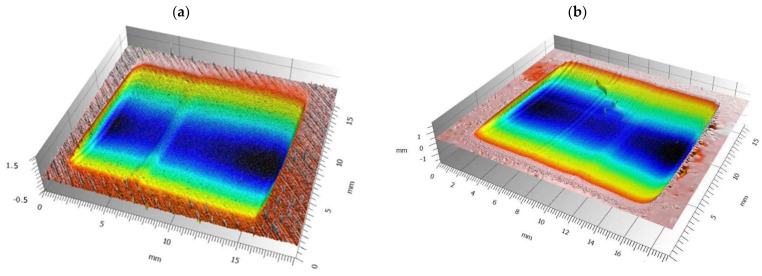
The surface topography after the abrasion wear test of a PET-G sample made by: (**a**) 3D printing; (**b**) extrusion. The color of the surface indicates the depth at which it is located. The colors in order from smallest to greatest depth: red, yellow, green, light blue, blue, dark blue.

The examination of the specimen surface with a profilometer allowed the roughness parameter Sa to be determined (Figure 11). The roughness parameter Sa was defined for the surface of the specimen before the abrasion test (Table 8) as well as for the wear scar surface after the test (Table 9). No correlation between the surface Sa parameter and the fabrication method of the sample was found. For ABS and PP materials, the Sa values for printed and extruded samples are similar, for PET-G, PLA and HIPS the Sa of printed samples is higher than that of extruded samples, and for PC, PMMA and PA the Sa of printed samples is lower than that of extruded samples.

The correlation coefficient between volume loss and the value of the parameter Sa of the sample’s surface for the extruded samples was 0.55, whereas for the printed samples it was 0.36. It ought to be noted that for the extruded samples there is a correlation between sample surface roughness and abrasion resistance, whereas for the printed samples there is no such significant correlation.

Similar observations were noted in studies on the abrasion sensitivity of engineering polymers and biocomposites, where the effect of surface roughness on abrasion resistance depended on the type of material and test conditions [32].

The correlation coefficient between volume loss and the value of the parameter Sa of the wear scar surface for extruded samples was 0.52, and for printed samples it was 0.45. No significant correlation between wear scar surface roughness and abrasion resistance was observed.

The correlation coefficient between hardness and roughness of the wear scar surface (the value of the parameter Sa) for extruded samples was 0.18, and for printed samples it was 0.52. For extruded samples with a homogeneous and compact structure, the effect of hardness on the roughness of the wear scar surface is low. For 3D printed samples, this effect is not significant but is slightly noticeable. It is possible that the structure of 3D prints consisting of paths and layers intensifies the effect of hardness on wear.

### 3.5. Microscopic Observations

**Figure 12:**
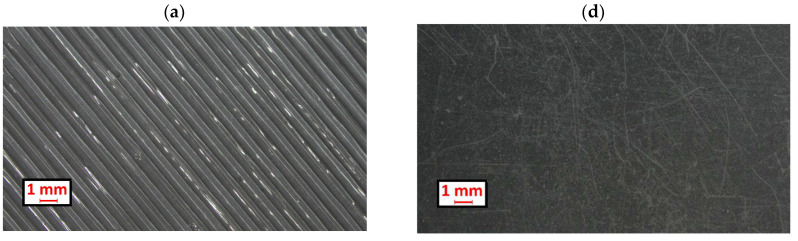

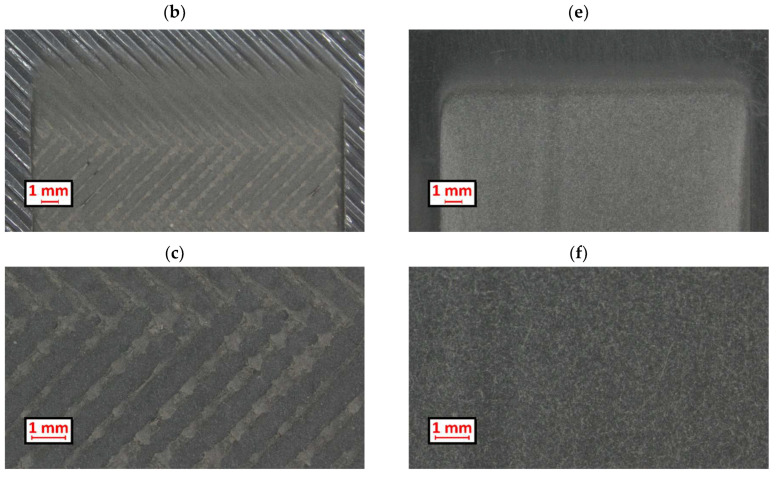
The surface of the 3D printed sample (**a**–**c**) and the extruded sample (**d**–**f**) before the abrasion wear test (**a**,**d**) and after the test (**b**,**e**) at 10× magnification. In addition, the abrasion surface was visualised at 20× magnification (**c**,**f**).

Despite printing at full fill (100%), the surface of the printed sample showed clear traces created by the movement of the nozzle (Figure 12a). The wear scar that could be observed on the sample revealed existing layers inside the sample, on which the paths applied by the nozzle are also clearly visible (Figure 12b,c). In the case of the extruded sample, only minor scratches from the cutting of the samples from the plates are visible on the surface (Figure 12d). The observed wear scar revealed a compact and homogeneous structure within the material (Figure 12e,f).

**Figure 13:**
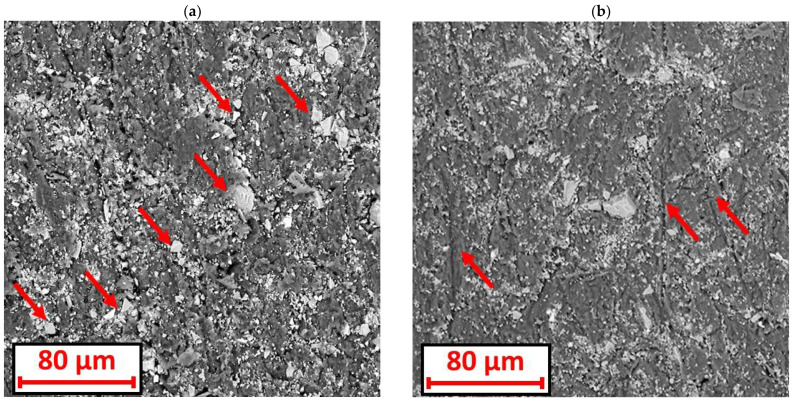
Wear scar at 1000× magnification: (**a**) the extruded ABS sample showing a large number of electrocorundum particles pressed into the plastic (a few selected particles are indicated by arrows); (**b**) the extruded PMMA sample showing scratches (indicated by arrows) oriented parallel to the direction of the rubber rim rotation.

The analysis of the images obtained with the SEM microscope appears to confirm the influence of hardness on the abrasive wear process (a clear correlation between microhardness and abrasion resistance was found for extruded samples, Section 3.4—hardness). The images of extruded samples with a higher hardness had significantly different characteristics on the surface than those with a lower hardness. For the extruded plastics of a lower hardness (ABS, PLA), while observing the wear scar, more electrocorundum grains were observed pressed into the plastic (Figure 13a) than for the samples of a higher hardness (Figure 13b) (PC, PMMA, PET-G, PA, HIPS). For the samples with a higher hardness, clearly visible scratches oriented parallel to the direction of rotation of the rubber rim appeared on the wear scar. Moreover, the pressed electrocorundum particles were smaller in size. It is likely that due to the significant hardness of the surface, the electrocorundum particles did not penetrate the material as easily, but were first moved and worked around it. As a result, the particles fragmented and left the scratches.

**Figure 14:**
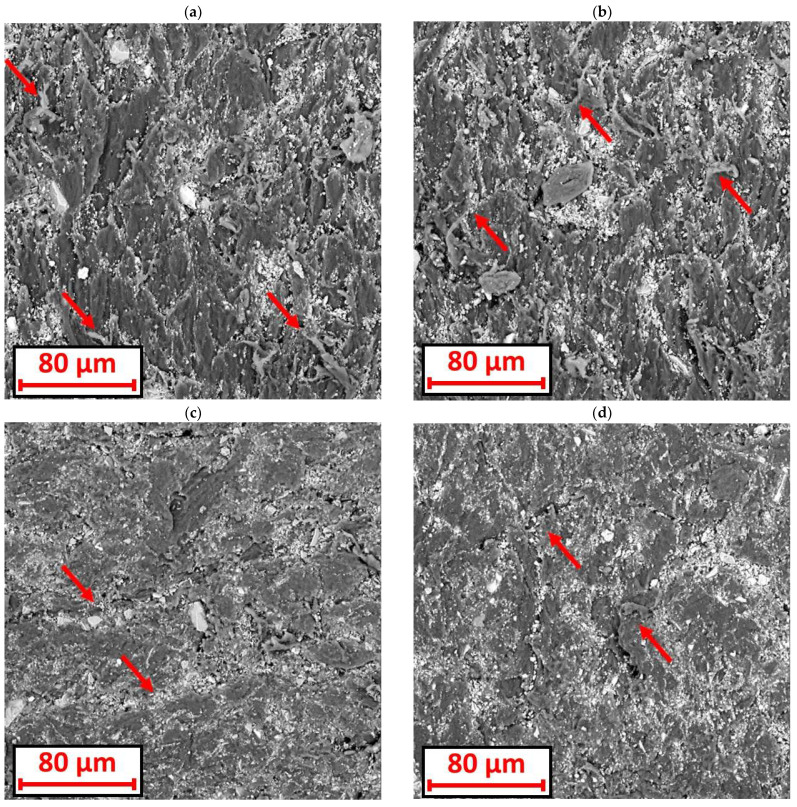
Wear scar at 1000× magnification: (**a**,**b**) the sample from printed PA with visible large number of “threads, fringes” (indicated by arrows); (**c**) the sample from printed PET-G with visible scratches (indicated by arrows) oriented perpendicular to the direction of the rubber rim rotation; (**d**) the sample from printed PET-G with visible pressing of the material (indicated by arrows).

The surfaces of the wear scar for the 3D-printed specimens looked significantly different from those of the extruded specimens. For the extruded specimens, a surface with no detachments from the material was observed. On the other hand, for printed samples, surface deformation was observed. It was in the form of detached material particles resembling threads protruding above the wear scar surface (Figure 14a,b). Such a deformation most likely appeared as the result of a material delamination. It is very possible that larger fragments of material were detached during the wear process. Furthermore, it is also possible that there were connections between the specimen and the piece being torn off. Connections might be torn apart after the piece has been completely detached, leaving fragments in the form of the previously mentioned threads. The shape of the deformation depends on the irregularities and the size of the abrasive particles.

Scratches oriented perpendicular to the direction of the roll rotation were observed on the surface of the wear scar. In terms of the printed specimens, the scratches were oriented parallel to the direction of the rim rotation. The grooves between the paths of the applied material (shown in the optical microscope images—Figure 12a) were oriented at an angle of 45° to the direction of the roll rotation. It is likely that the abrasive fell into these grooves and was guided through them, resulting in a perpendicular scratch.

On the surface of the wear scar for the printed samples, “pressed, smushed” fragments of plastic were also visible (Figure 14c,d). It is probable these fragments had become detached from the sample and were later pressed into the surface by the rubber rim.

### 3.6. Young’s Modulus

**Figure 15:**
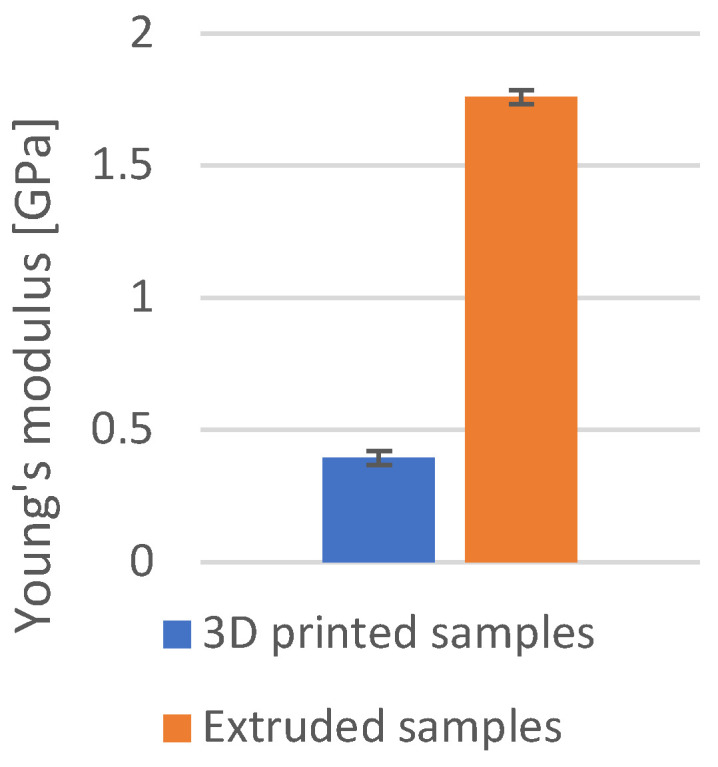
Young’s modulus for the extruded and the printed PP.

Young’s modulus tests were carried out only for the PP, as only in this case was a noticeably significant difference in the stiffness of the printed and extruded samples observed. The beam deflection tests show that the Young’s modulus of the printed PP is 0.395 ± 0.027 GPa, which is more than four times lower than the Young’s modulus of the extruded PP of 1.760 ± 0.026 GPa (Figure 15).

## 4. Discussion

The tested plastics significantly differed from each other and presented a wide spectrum of properties. Despite these differences, it can be stated that extruded samples are more resistant to abrasive wear than 3D-printed samples (six out of the eight extruded materials appeared to be more resistant to abrasive wear). The first part of the discussion tries to explain this phenomenon. The second part focuses on the materials for which extruded samples are less resistant to abrasive wear.

The extruded samples are from 9% to 22% more abrasion-resistant than the printed ones. The abrasion resistance of the extruded samples is dependent on factors reported in the literature such as hardness, density and surface roughness (proved by the values of the correlation coefficients). In the case of 3D-printed samples, no such correlation was found (proved by the values of the correlation coefficients). For this reason, the researchers of the current study believe that the explanation for the reduced abrasion resistance of printed samples should be sought by analysing their internal structure.

On the basis of the surface topography measurements, it may be stated that the surface of the 3D-printed specimens is quite similar to that of the extruded materials, as quite similar roughness values were obtained after measurements. However, macroscopically, grooves parallel to each other are present on the surface of the printed samples. Such grooves are a result of laid paths of molten material. It is possible that at the beginning of the interaction between the abrasive and the sample, the abrasive material catches on the surface with greater intensity as a result of the presence of these grooves.

The interior of the printed sample was also made up of layered parallel paths. When one layer was removed as a result of wear, another layer was exposed, on which the grooves were also present. Most likely, the internal inhomogeneity of the samples is another factor that increases the intensity of abrasive wear.

Subramaniyan et al. [24] indicate that even at a 100% fill, there are wear-increasing voids in the printed sample, and the layered structure of the print promotes layer delamination and increased wear.

The 3D-printed parts may have lower strength than the extruded parts [25]. Furthermore, the strength of 3D prints is dependent on the orientation of the force direction relative to the direction of the applied paths [26]. It is also very likely that during abrasive wear, fragments of the 3D-printed samples detach more easily in comparison to the extruded samples.

Extruded samples are from 9% to 22% more abrasion-resistant than printed samples.

An extruded PA sample was 75% more abrasion-resistant than a printed sample (for the other plastics tested, this difference is in the range of 9% to 22%). It is important to explain why there is such a large difference. In the case of polyamide, polyamide 12 was tested for printed samples and polyamide 6 for samples cut from extruded sheets. These materials belong to the same group, but differ in chemical structure and properties. For the production of plates, PA6 is used overwhelmingly; plates with PA12 are rare. When producing filaments for FDM 3D printers, the opposite is true: PA12 is by far the predominant material and PA6 is rare. Moreover, it is likely that the difference in PA grade is the reason for the variation in wear scar geometry (Section 3.1. abrasion resistance, Figure 8).

As the main aim of the study was to indicate whether 3D printing or prefabricated parts are better to use for the production of a component, it was decided to compare samples printed from PA12 against samples extruded from PA6. Such a comparison ought to be more beneficial for 3D printer users, as it will help them decide whether to purchase a filament or a prefabricated part. Comparing the same grades of polyamide (PA6 plate versus PA6 print and PA12 plate versus PA12 print) would have made the research more scientific, but would have deprived it of the practical applications that the authors cared more about.

The previously described regularity with which extruded samples were more resistant to abrasive wear than printed ones is not true for PP and HIPS. In the case of PP, a significant difference in the stiffness of the printed (Young’s modulus is 0.395 ± 0.027 GPa) and extruded (Young’s modulus is 1.760 ± 0.026 GPa) specimen was observed. Furthermore, the difference in stiffness became apparent during hardness measurements. When tested by the Vickers method for PP, the printed impression was not observed. During indentation, the material deflected and returned to its original shape when the indenter was withdrawn. For extruded PP, the imprint was visible. For this reason, the hardness of the PP was measured using a Shore D hardness tester. It is most likely that when the abrasive rolls over the surface of the printed PP, the lower stiffness causes the material to deflect and this minimises the abrasive catching on the sample. Extruded PP is not as flexible and, thus, the abrasive catches on the material more easily and causes more intense specimen weight loss.

The difference in PP stiffness most likely contributes to the different shape of the wear scar for printed and extruded PP (Section 3.1. abrasion resistance, Figure 8). The less stiff printed PP lays down more freely under the rubber rim, which results in more varied wear scar geometry. The stiffer extruded PP gives a more regular, cylindrical wear scar shape.

The difference in the hardness and stiffness of the printed and the extruded PP samples may be due to differences in processing parameters, which leads, among other things, to differences in the crystallization process and its evolution [27].

HIPS is a polymer blend produced by in situ polymerisation of styrene in solution with polybutadiene rubber. Polybutadiene can have different particle configurations in the polymer chain, and as indicated by Rovere et al. it has strong influence on the mechanical properties [28]. Material manufacturers very rarely provide information on the form of polybutadiene used in a given HIPS, and sometimes it is even a company secret. It is highly possible that the HIPS used for extruding the sheets and the HIPS used for 3D printing differ in the polybutadiene used, which affected the mechanical properties to such an extent that different abrasion resistance was obtained.

## 5. Conclusions

This paper presents a comparison of the abrasion resistance of FDM 3D prints and extruded materials. The abrasion resistance of 3D prints and extruded samples has already been analysed [28,29,30]; however, these materials were analysed separately and their comparison was not available. The research presented in this paper has shown that almost all of the extruded samples (for six out of the eight materials tested) were more resistant to abrasive wear than the printed ones (between 10% and 24%).

The abrasion resistance of samples made by extrusion technology is dependent on factors reported in the literature, such as hardness, density and surface roughness. In the case of the 3D-printed samples, no such relationship was found. This is proved by the values of the correlation coefficients. For this reason, the researchers believe that the reduced abrasion wear resistance of printed samples is due to their internal structure.

In some cases, fabrication by the 3D printing led to a higher abrasion wear resistance (HIPS and PP) or was significantly different from the value obtained for the extruded sample (almost 75% difference for the PA). A material for 3D printing can be described by the same name but exhibit different properties. For example, in the case of PA, a different type of this material was used in 3D printing (PA6 for extrusion and a mixture of PA6 and PA 12 for 3D printing). Very often, material manufacturers simply call the material polyamide, and details about the polyamide types used can only be found in the detailed data. In the case of parts exposed to abrasive wear, when aiming to produce with FDM 3D printing a component that was previously made with extrusion technology, it should be verified that the filament used for 3D printing has the same properties as the material after extrusion.

In terms of elements that are exposed to abrasion, polymer-based composites are very often used. Plastics are reinforced with, among others, carbon or glass fibres, as well as graphite powders or molybdenum disulfide powders. In the next stages of research, it would be worth carrying out measurements for composites.

## Figures and Tables

**Table 1:** The details of the materials used to print the samples and the most important parameters of the printing process.

Material	Nozzle Temperature [°C]	Bed Temperature [°C]	Trade Name	Manufacturer
PLA	205	40	Z-PLA	Zortrax
PET-G	225	40	Z-PETG	
ABS	250	95	Z-ABS	
PA12	260	95	Z-NYLON	
PC	270	105	PC-IN	F3D Filament
PMMA	270	105	PMMA	
HIPS	240	95	HIPS	
PP	240	80	PP	Fiberlogy

**Table 2:** The most important process parameters during the sample printing.

Nozzle diameter	0.4 mm
Layer height	0.2 mm
Density of infill	100% (full)
Orientation of the infill path in relation to the edge of the sample	45°
Mutual orientation of the infill path of adjacent layers	90°
Number of tracks arranged on the perimeter (contour)	3
Contour—filling gap	0 mm (no gap)

**Table 3:** Detailed parameters of the test apparatus and abrasion resistance measurement.

Counter-Sample Counter-Sample Rotational Speed	Rubber Lined Wheel ø50 mm × 15 mm (Hardness 78 ÷ 85°ShA) 60 rpm
Number of counter-sample rotations	600
Pressure force of the sample against the counter-sample	44N
Sample dimensions	30 mm × 30 mm × 2 mm
Abrasive	Electrocorundum No. 90 (PN-76/M-59115)

**Table 4:** Volume loss for the extruded sample with respect to the result obtained for the printed sample.

ABS	PC	PMMA	PET-G	PLA	PA	HIPS	PP
−14.4% (−9.7% ÷ −18.8%)	−19.5% (−13.1% ÷ −25.2%)	−21.6% (−9.9% ÷ −25%)	−9.6% (−4.3% ÷ −12.8%)	−13.3% (−3.9% ÷ −19.7%)	−74.6% (−31.5% ÷ −91.6%)	16.3% (10.6% ÷ 29.2%)	34.1% (10.8% ÷ 121.2%)

**Table 5:** Wear scar depth for the extruded sample with respect to the result obtained for the printed sample.

ABS	PC	PMMA	PET-G	PLA	PA	HIPS	PP
−10.1% (−18.7% ÷ 0.2%)	−9.5% (−2.5% ÷ −16.0%)	−1.5% (−7.0% ÷ 4.3%)	5.0% (−3.0% ÷ 13.9%)	−29.6% (−28.0% ÷ −31.2%)	−27.8% (−12.5% ÷ −41.5%)	37.7% (32.4% ÷ 43.0%)	77.2% (59.9% ÷ 98.0%)

**Table 6:** The density of the extruded sample with respect to the result obtained for the printed sample.

ABS	PC	PMMA	PET-G	PLA	PA	HIPS	PP
29.6% (22.2% ÷ 38.0%)	38.8% (28.9% ÷ 48.9%)	36.9% (29.5% ÷ 44.6%)	23.0% (17.3% ÷ 28.9%)	3.5% (−1.8% ÷ 8.8%)	34.8% (25.5% ÷ 45.1%)	4.0% (3.1% ÷ 5.0%)	0.0% (−6.0% ÷ 6.6%)

**Table 7:** Changes in the microhardness/hardness value of an extruded sample with respect to the result obtained for the printed sample.

ABS	PC	PMMA	PET-G	PLA	PA	HIPS	PP
−7.3% (17.5% ÷ −26.2%)	258.2% (199.4% ÷ 335.5%)	9.4% (−8.8% ÷ 34.2%)	175.4% (126.9% ÷ 236.6%)	16.5% (−9.3% ÷ 48.4%)	18.5% (−1.1% ÷ 44.7%)	41.3% (18.1% ÷ 67.6%)	63.0% (54.7% ÷ 72.2%)

**Table 8:** The Sa parameter for the sample’s surface.

Type of Samples	ABS	PC	PMMA	PET-G	PLA	PA	HIPS	PP
3D printed samples [µm]	21.3	0.1	21.9	32.3	32.6	0.1	28.5	7.7
Extruded samples [µm]	12.0	3.6	26.4	7.8	41.7	15.3	17.2	9.8

**Table 9:** The Sa parameter for the wear scar.

Type of Samples	ABS	PC	PMMA	PET-G	PLA	PA	HIPS	PP
3D printed samples [µm]	19.9	6.5	35.2	12.8	18.4	12.9	13.1	7.8
Extruded samples [µm]	12.3	9	15.2	13.2	21.7	21.2	16.8	11.4

## Data Availability

The original contributions presented in this study are included in the article. Further inquiries can be directed to the corresponding author.

## References

[1] (2025). Grand View Research 3D Printing Market Size & Trends Market Concentration & Characteristics. https://www.grandviewresearch.com/industry-analysis/3d-printing-industry-analysis#.

[2] Baumann F., Roller D. (2016). Vision based error detection for 3D printing processes. MATEC Web Conf..

[3] Verhoef L.A., Budde B.W., Chockalingam C., García Nodar B., van Wijk A.J.M. (2018). The effect of additive manufacturing on global energy demand: An assessment using a bottom-up approach. Energy Policy.

[4] Huang R., Riddle M., Graziano D., Warren J., Das S., Nimbalkar S., Cresko J., Masanet E. (2016). Energy and emissions saving potential of additive manufacturing: The case of lightweight aircraft components. J. Clean. Prod..

[5] Atzeni E., Salmi A. (2012). Economics of additive manufacturing for end-usable metal parts. Int. J. Adv. Manuf. Technol..

[6] Atzeni E., Iuliano L., Minetola P., Salmi A. (2010). Redesign and cost estimation of rapid manufactured plastic parts. Rapid Prototyp. J..

[7] Çevik Ü., Kam M. (2020). A review study on mechanical properties of obtained products by FDM method and metal/polymer composite filament production. J. Nanomater..

[8] Kampker A., Bergweiler G., Hollah A., Lichtenthäler K., Leimbrink S. (2019). Design and testing of the different interfaces in a 3D printed welding jig. Procedia CIRP.

[9] Krznar N., Pilipović A., Šercer M. (2016). Additive manufacturing of fixture for automated 3d scanning—Case study. Procedia Eng..

[10] Chitariu D.-F., Munteanu A. (2018). Research on 3D printed fixture components. MATEC Web Conf..

[11] Elkholy A., Kempers R. (2020). Enhancement of pool boiling heat transfer using 3D-printed polymer fixtures. Exp. Therm. Fluid Sci..

[12] Wahab Hashmi A., Singh Mali H., Meena A. (2022). Design and fabrication of a low-cost one-way abrasive flow finishing set-up using 3D printed parts. Mater. Today Proc..

[13] Roudný P., Syrový T. (2022). Thermal conductive composites for FDM 3D printing: A review, opportunities and obstacles, future directions. J. Manuf. Process..

[14] Faidallah R.F., Hanon M.M., Szakál Z., Oldal I. (2022). Biodegradable materials used in FDM 3D printing technology: A critical review. J. Mod. Mech. Eng. Technol..

[15] Sztorch B., Brząkalski D., Pakuła D., Frydrych M., Špitalský Z., Przekop R.E. (2022). Natural and synthetic polymer fillers for applications in 3D printing—FDM technology area. Solids.

[16] Ahmed W., Alnajjar F., Zaneldin E., Al-Marzouqi A.H., Gochoo M., Khalid S. (2020). Implementing FDM 3D printing strategies using natural fibers to produce biomass composite. Materials.

[17] Manoj A., Panda R.C. (2022). Biodegradable filament for 3D printing process: A review. Eng. Sci..

[18] Kristiawan R.B., Imaduddin F., Ariawan D., Ubaidillah, Arifin Z. (2021). A review on the fused deposition modeling (FDM) 3D printing: Filament processing, materials, and printing parameters. Open Eng..

[19] Doshi M., Mahale A., Kumar Singh S., Deshmukh S. (2022). Printing parameters and materials affecting mechanical properties of FDM-3D printed Parts: Perspective and prospects. Mater. Today Proc..

[20] Fouly A., Assaifan A., Alnaser I., Hussein O., Abdo H. (2022). Evaluating the mechanical and tribological properties of 3D printed polylactic-acid (PLA) green-composite for artificial implant: Hip joint case study. Polymers.

[21] Hanon M.M., Zsidai L. (2021). Comprehending the role of process parameters and filament color on the structure and tribological performance of 3D printed PLA. J. Mater. Res. Technol..

[22] Palaniandy L., Ismail K.I., Yap T.C. (2023). Tribological behaviour of 3D printed polylactic acid (PLA) sliding against steel at different sliding speed. J. Phys. Conf. Ser..

[23] Hanon M.M., Alshammas Y., Zsidai L. (2020). Effect of print orientation and bronze existence on tribological and mechanical properties of 3D-printed bronze/PLA composite. Int. J. Adv. Manuf. Technol..

[24] Maries I.T., Vilau C., Pustan M.S., Dudescu C., Crisan H.G. (2020). Determining the tribological properties of different 3D printing filaments. IOP Conf. Ser. Mater. Sci. Eng..

[25] Mitaľ G., Gajdoš I., Spišák E., Majerníková J., Jezný T. (2022). An analysis of selected technological parameters’ influences on the tribological properties of products manufactured using the FFF technique. Appl. Sci..

[26] Unal H., Sen U., Mimaroglu A. (2005). Abrasive wear behaviour of polymeric materials. Mater. Des..

[27] Leśniewski T., Wieleba W., Krawczyk J., Biernacki K., Opałka M., Aldabergenova T. (2024). Tribological properties of thermoplastic elastomer used in 3D printing technology. Aviation.

[28] Ercegović Ražić S., Sutlović A., Godec D., Kutnjak-Mravlinčić S. (2024). Testing the abrasion resistance of 3D printed test specimens made of acrylonitrile/butadiene/styrene by fused deposition modeling. Koža Obuća.

[29] Ol’khovik E. (2017). Study of abrasive resistance of foundries models obtained with use of additive technology. IOP Conf. Ser. Earth Environ. Sci..

[30] Suresha B., Hanamasagar V., Jamadar I.M., Arvind S.L., Somashekar H.M. Mechanical properties and abrasion resistance of 3D printed lightweight cf-reinforced PLA/ABS composites using design of experiments. Proceedings of the International Symposium on Lightweight and Sustainable Polymeric Materials.

[31] Rajesh J.J., Bijwe J., Tewari U. (2002). Abrasive wear performance of various polyamides. Wear.

[32] Muhandes H., Kalácska Á., Székely L., Keresztes R., Kalácska G. (2020). Abrasive sensitivity of engineering polymers and a bio-composite under different abrasive conditions. Materials.

